# Gene expression profiling of melanoma cells – searching the haystack

**DOI:** 10.1186/1479-5876-3-2

**Published:** 2005-01-13

**Authors:** Patricia Brafford, Meenhard Herlyn

**Affiliations:** 1 The Wistar Institute, Philadelphia PA 19104, USA

## Abstract

Cancer is being increasingly recognized as a very heterogeneous disease, both within an individual tumor and within a tumor type and among tumor types. This heterogeneity is manifested both at the genetic and phenotypic level and determines the progression of disease and response to therapy. It is possible to see the heterogeneity in examples of differential disease progression and response to therapy of the same tumor type, as morphology does not always reveal underlying biology. The diagnosis of tumors by histopathological and morphological criteria cannot fully account for the variability seen in prognosis and therapy outcome. Here we review some recent concepts that have emerged from the genetic analysis of metastatic melanoma.

## Commentary

Cancer is being increasingly recognized as a very heterogeneous disease, both within an individual tumor and within a tumor type and among tumor types. This heterogeneity is manifested both at the genetic and phenotypic level and determines the progression of disease and response to therapy. It is possible to see the heterogeneity in examples of differential disease progression and response to therapy of the same tumor type, as morphology does not always reveal underlying biology. The diagnosis of tumors by histopathological and morphological criteria cannot fully account for the variability seen in prognosis and therapy outcome. A classic example of this heterogeneity is diffuse large B cell lymphoma (DLBCL) morphologically defined as one tumor type but only 40% of patients respond to treatment suggesting there are at least two distinct tumor groups. Alizadeh et. al. performed microarrays on DLCBL to assess gene expression profiles and identified several genetically distinct groups that correlated with differential survival rates [[1], reviewed in [2]]. Genome wide screening technology such as microarray offer the potential to diagnose, prognose and develop new therapeutic strategies for cancers based on grouping by genetic signature.

Whereas previously, it was possible only to study one or a few genes at a time, microarray technology allows the simultaneous assessment of the expression of thousands of genes within a cell population at a single time. By looking at the full spectrum of the genetic contribution within a tumor, microarray technology has furthered our understanding of the complexity in terms of tumor subclassification. The advantage of a global gene expression analysis is that it assesses many genes within a sample at a given timepoint and allows comparison to a myriad of other samples. This has resulted in the improved classification of tumors, identification of potential new biomarkers, and detection of possible therapeutic targets as in DLBCL. In addition, the same gene expression data can be reanalyzed according to a user defined phenotype or without bias while looking for patterns in the data that correlate with a phenotype such as progression, prognosis, or treatment outcome. As data analysis and data mining become more sophisticated, the information acquired will provide scientists and clinicians with a significant improvement in correlating patient data with tumor diagnosis and enabling us to better select patient groups who will respond (or not respond) to therapy. Microarray technology has become the best hope in developing a global and accurate assessment of the tumor type and all its complexity. However, the road to achieve this goal will be long and hard because we have to learn to ask the right questions, select the appropriate patients, collect their material and then verify the initial results.

Clinical pathological analysis cannot predict clinical outcome or metastatic potential of melanoma, a very heterogeneous cancer with an unpredictable progression rate. In a previous issue, Wang et. al. performed gene expression analysis on RNA from a number of human solid tumor lesions, including melanoma [3]. In their comparison they showed that it is possible to identify tumor specific gene expression profiles which can rapidly aid in tumor identification and classification. In addition, the study identified commonly expressed genes between melanoma and Renal Cell carcinoma, both known to be responsive clinically to IL-2 treatment, allowing for comparison of immunologically related genes to identify common response pathways. Gene expression profiles of melanoma lesions can also be used for prognosis by stratifying patients based on risk and thus identifying subtypes. For example, an early study by Clark et. al. assessed the gene expression differences of metastatic versus non-metastatic melanoma cell lines, identifying a metastatic profile which was linked to the small GTPase RhoC [4]. Bittner et. al. performed a more comprehensive array analysis of 31 cutaneous melanomas and identified a major cluster of melanoma samples [5]. Further, the authors were able to verify the validity of the cluster by correlating the melanomas within the cluster to reduced motility, invasive ability, and vasculogenic mimicry potential *in vitro*. This showed that lesions can be stratified into subtypes by gene expression analysis. Further, microarray gene expression data can be used to define responders and non-responders to known anti-cancer treatments prospectively or retrospectively. By combining clinical data with microarray data, it will be possible to predict patient response based on gene expression profile or biomarkers, which may allow for better, more targeted therapies to be selected. This new information will lead to improved treatments and prolonged survival for cancer patients.

DNA microarray technology may help us understand the complex pathogenesis of melanoma and will allow us to determine the role of the different genetic profiles in determining different disease outcomes. From this we will be able to identify new biomarkers, leading to the development of more pathologically relevant models. To achieve better prediction for optimal treatment strategies, microarray studies as presented here are only the beginning of a long road, in which we need to drastically par down the markers to be tested. We need to verify and validate biomarker candidates in ways that go beyond the capacity of individual laboratories. Instead we need to establish consortia of scientists from bioinformatics and computational biology, who team up with oncologists, pathologists, and immunobiologists. Any selected biomarker requires validation in independent multi-center analyses. Once the appropriate tools and infrastructures are on hand, we can select better new treatment modalities and may realize that previously unsuccessful regimens would have shown more success, if we would have know how to select most appropriate patients. We have to start now to develop the groundwork for such multidisciplinary, multi-institutional work that will challenge us in the years to come.
